# Metal-Organic Framework (MOF)/Epoxy Coatings: A Review

**DOI:** 10.3390/ma13122881

**Published:** 2020-06-26

**Authors:** Farzad Seidi, Maryam Jouyandeh, Mohsen Taghizadeh, Ali Taghizadeh, Henri Vahabi, Sajjad Habibzadeh, Krzysztof Formela, Mohammad Reza Saeb

**Affiliations:** 1 Provincial Key Lab of Pulp and Paper Science and Technology and Joint International Research Lab of Lignocellulosic Functional Materials, Nanjing Forestry University, Nanjing 210037, China; f_seidi@njfu.edu.cn; 2 Center of Excellence in Electrochemistry, School of Chemistry, College of Science, University of Tehran, Tehran 11155-4563, Iran; maryam.jouyande@gmail.com (M.J.); taghizadeh.mohsen1995@gmail.com (M.T.); ali_taghizadeh1995@yahoo.com (A.T.); 3 Université de Lorraine, CentraleSupélec, LMOPS, F-57000 Metz, France; henri.vahabi@univ-lorraine.fr; 4 Department of Chemical Engineering, Amirkabir University of Technology (Tehran Polytechnic), Tehran 15916-39675, Iran; sajjad.habibzadeh@aut.ac.ir; 5 Department of Polymer Technology, Faculty of Chemistry, Gdańsk University of Technology, Gabriela Narutowicza 11/12, 80-233 Gdańsk, Poland; 6 Department of Resin and Additives, Institute for Color Science and Technology, Tehran P.O. Box: 16765-654, Iran

**Keywords:** metal-organic framework (MOF), epoxy, coating, anti-corrosion performance, flame retardancy, mechanical properties

## Abstract

Epoxy coatings are developing fast in order to meet the requirements of advanced materials and systems. Progress in nanomaterial science and technology has opened a new era of engineering for tailoring the bulk and surface properties of organic coatings, e.g., adhesion to the substrate, anti-corrosion, mechanical, flame-retardant, and self-healing characteristics. Metal-organic frameworks (MOFs), a subclass of coordinative polymers with porous microstructures, have been widely synthesized in recent years and applied in gas and energy storage, separation, sensing, environmental science and technology, and medicine. Nevertheless, less attention has been paid to their performance in coatings. Well-known as micro- and nanoporous materials, with a tailorable structure consisting of metal ions and organic linkers, MOFs have a huge loading capacity, which is essential for the delivery of corrosion inhibitors. This review paper attempts to highlight the importance of epoxy/MOF composites for coating applications. A particular emphasis was explicitly placed on the anti-corrosion, flame-retardant, mechanical, and dielectric properties of epoxy/MOF coatings.

## 1. Introduction

Protective coatings can be found everywhere, in a wide range of applications, from sports floor coatings to anti-corrosive coatings for use in aerospace [1,2]. Organic coatings are mainly thermoset resins, designed for the protection of a substrate, frequently metals, from corrosion or a corrosive media [3,4]. Nevertheless, due to the high life expectancy nowadays, multifunctional organic coatings with an all-in-one character are in demand [5,6,7,8]. Nanoparticles of different families, sizes, shapes, and surface functionalities have been used in developing advanced organic coatings [9,10,11,12]. Although there is a choice of several different nanoparticles, researchers are looking for those possessing the functional groups that are reactive towards resins and/or curing agents for high-performance requirements [13,14,15]. Particular attention has been directed towards porous minerals with optimum pore size and pore size distribution potent to post-functionalization [16,17,18]. To tackle the inadequate properties of coatings, developing coatings that are reinforced with nanoporous materials as capsules or containers has become a hot area of research. The conventional nanomaterials, such as carbon nanotube, graphene oxide, nano-silica, layered double hydroxide (LDH), and polyhedral oligomeric silsesquioxane (POSS) capable of being encapsulated, have been extensively examined for organic coatings [19,20,21]. However, the synthesis of organic–inorganic hybrid minerals with a high potential for interaction with polymer matrices has received much more attention.

**Figure 1:**
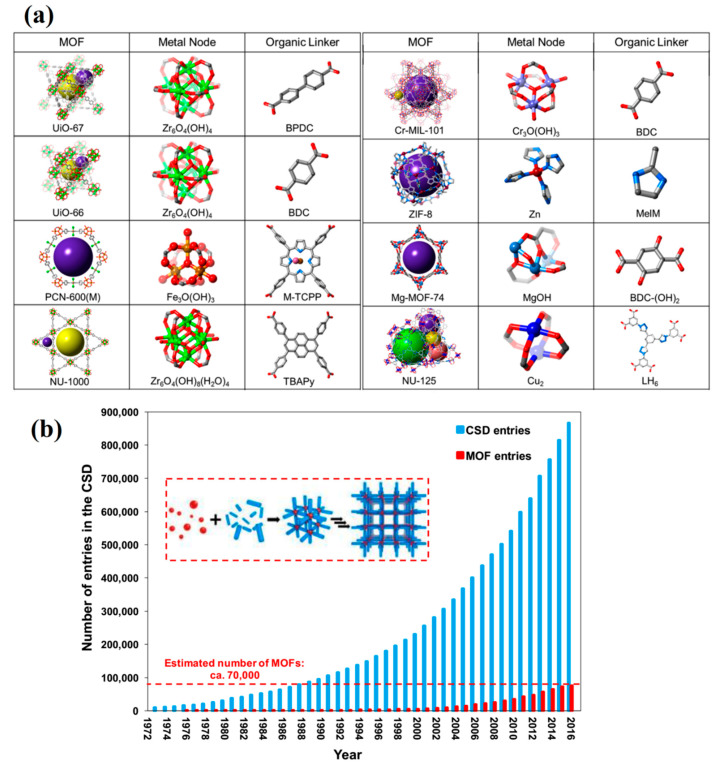
Metal-organic framework (MOF); (**a**) combination of building blocks of MOF, with different types of metal atoms as the nodes and various kinds of carboxylic ligands as the organic linkers, used in the fabrication of exquisite structures [39], (**b**) The number of different kinds of fabricated MOF structures (Source: The Cambridge Structural Database (CSD)) [64] revealing that more than 70,000 different structures of MOFs have been introduced prior to 2016. The exponential trend in the number of publications released in the last decade extracted from the Scopus database (based on the keywords of “metal-organic framework” and “MOF”) can be observed.

Porous coordinative polymers (PCPs), well-known as metal-organic frameworks (MOFs), are novel organic–inorganic, highly porous structures that are obtained through precisely controlled structures in which two components of metal cations as nodes and organic molecules as bridges have been engineered, as shown in Figure 1a [22,23,24]. The broad range of complex metal cations and connector molecules on one side, and the flexibility of synthesis routes along with the ability for post-functionalization of such structures on the other side, give rise to a synthesis of the bewildering arrays of possible MOFs with 2D or 3D structures [25,26,27]. Recently, mixed-metal MOFs and reproducible bio-MOFs have been obtained via the disciplined mixing of several metal cations with biomolecules, e.g., peptides, instead of conventional organic linkers [28,29]. The exceptional characteristics of MOFs, including a very large surface area (theoretically even up to 14,600 m^2^/g) [30], permanent porosity, tailorable pore size and pore size distribution, chemical versatility, architectural flexibility, ease of functionalization, and high mechanical and thermal stability, render them superior to other porous materials. Moreover, some MOF complexes additionally possess antimicrobial properties, self-cleaning abilities, and easy recoverability [31]. As a result, there has been an almost exponential trend in the number of papers published on MOFs in the past few years, as shown in Figure 1b.

**Figure 2:**
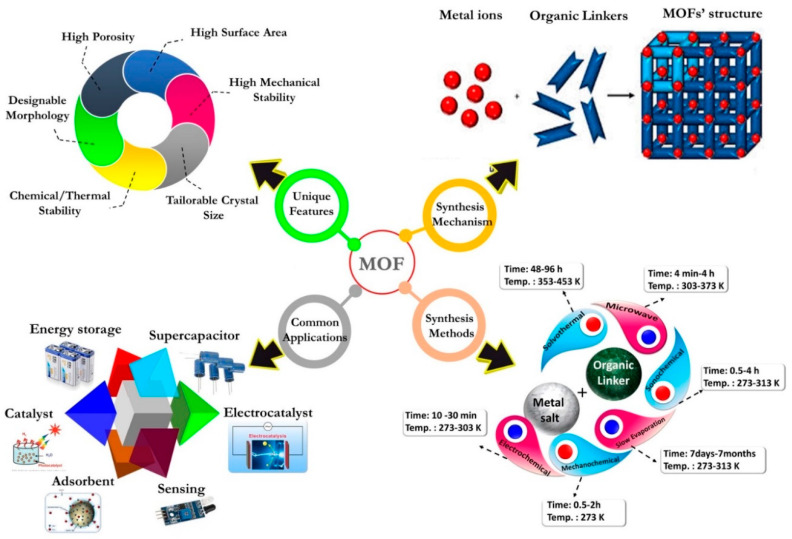
Schematic illustration of the major synthesis routes from solvothermal and microwave to electrochemical techniques with different ranges of temperature and fabrication time applied in MOF synthesis; unique features of MOFs such as chemical/thermal stability, designable morphology, tailorable crystal size, large surface area, and excellent porous structure; typical applications of MOFs such as energy storage, supercapacitors, sensing, and catalysis; and the synthesis mechanism [7,13].

Different from other porous nanoparticles, MOFs are flexible to microstructural changes by manipulating the type and the amount of metal ions and the organic linkers [32,33,34]. MOFs can be synthesized into different forms, such as linear, square, planar, and triangular, with variable properties [35,36]. Di-, tri-, and tetravalent metal ions can be used as inorganic units in MOF structures [37]. Evidently, the data found in the literature reveals that the majority of MOFs with various structures and topologies have been fabricated by using different methods, such as the traditional solvothermal and non-solvothermal strategies [38,39,40]. Careful selection of appropriate metal ions and organic ligands allows for the production of architecturally optimized MOFs for high-tech applications [41]. Due to their tailorable microstructure and properties, MOFs have been extensively used for energy storage [42,43], sensing [44,45,46], environmental uses [47,48,49], and particularly in medicine [50,51]. In recent years, MOFs have appeared to be superior to some conventional porous materials, such as zeolite and activated carbons, as shown in Figure 2 [52].

**Figure 3:**
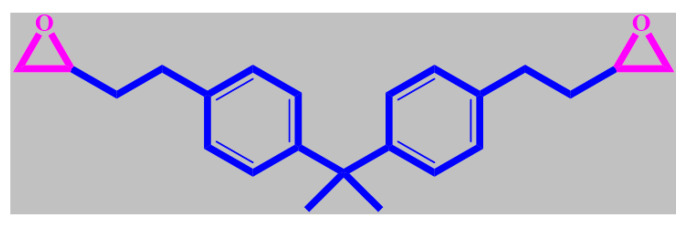
Chemical structure of diglycidyl ether bisphenol-A (DGEBA) epoxy resin.

Epoxy can react with polyfunctional curing agents, including amines, acids, phenols, alcohols and thiols, to form a cross-linked thermosetting polymer intended to develop coatings with outstanding mechanical properties, processability, high strength and modulus, and chemical resistance, as well as low creep, good electrical insulating properties, and high thermal stability [53,54,55]. Nevertheless, the prosperous application of epoxy coatings can be limited due to their susceptibility to damage by surface abrasion [56]. The poor resistance of epoxy resins against the initiation and propagation of cracks is another problem, which can impair their mechanical strength [57]. In addition, the hydrophilicity of epoxy causes high shrinkage during the curing process of epoxy, which facilitates water uptake from the surrounding environment [58]. The incorporation of porous MOFs in epoxy coatings has been recognized by some experts to compensate for their low properties. For example, the poor dispersion of nanoparticles in epoxy resin is a challenge in the preparation of epoxy nanocomposites [59]. It is believed that MOFs as a hybrid of organic and inorganic materials can take advantage of functional organic groups to make possible uniform dispersion in the epoxy matrix. The structure of the common epoxy resin, diglycidyl ether bisphenol-A (DGEBA), is shown in Figure 3. Moreover, the large specific surface areas of MOFs can guarantee a strong interfacial interaction between the epoxy matrix and the mineral.

Although the importance of MOFs in wide range of applications has been recognized, work on the different aspects of MOF-based epoxy coatings is still in the early stages of its development. Moreover, on the grounds of the above discussion, MOF/epoxy coatings can be taken as the next generation of high-performance versatile composite coatings. In this review article, a particular emphasis is placed on collecting data on MOF/epoxy systems which are applied as coatings. The privilege of this class of organic coatings lies in the ability to be multidisciplinary coatings whose properties can be tailored by manipulating their size, porosity, concentration, and curing circumstances [60,61,62,63]. The anti-corrosion, flame-retardant, mechanical, and dielectric properties of epoxy/MOFs coatings have been discussed in this regard. 

## 2. MOFs in Epoxy Coatings

**Figure 4:**
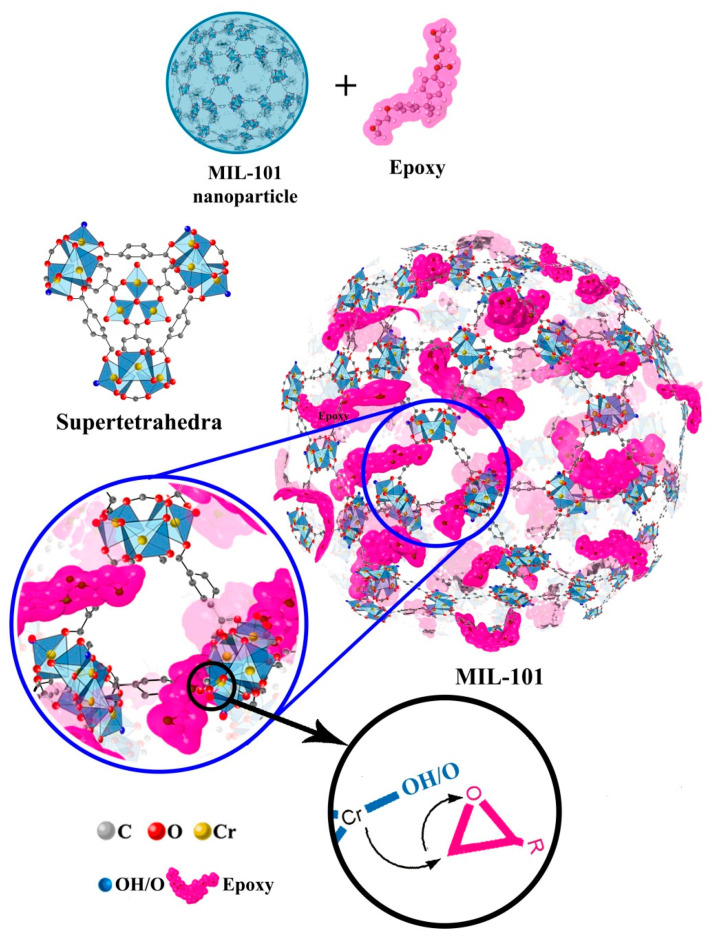
The possible reaction between the MIL-101 MOF and the epoxy resin and the schematic view of the diffusion of epoxy into the pores of MOF [70]. Similar to the outcome of studies performed on halloysite nanotubes (HNTs), using the potential of inner functional groups (in the case of HNTs, inside the nanotubes and in the case of MOF, inside the nanocages) has a significant effect on the crosslinking of epoxy resin. In fact, functional groups contributed to curing reactions from the internal surface of minerals (denoted as secondary curing agents or extra cure sites) keeping the stoichiometry balanced when gelation takes place. Above conversion of gelation (α_g_), curing agent molecules can hardly access the epoxide groups; therefore, curing will remain incomplete as a consequence of contacts between cure moieties being substantially limited or even stopped. In such a situation, the excess sites contributed from reactive groups of internal structure compensate for incomplete cure arising from gelation. MOF has a high potential for being considered as such a highly reactive mineral.

Due to the exquisite structure of MOFs, their surfaces can be further functionalized to make them reactive for developing MOFs/polymer nanocomposites. The presence of organic linkers in the structures of MOFs gives them the privilege of appearing compatible with the polymeric matrices [65,66]. Typically, MOFs can form an organic–inorganic hybrid structure, which can be appropriately dispersed within the polymer [67]. The introduction of highly porous MOF nanocages with desired topological textures into a thermoset polymer matrix such as epoxy can offer facile curing [68,69]. Jouyandeh et al. [70] demonstrated that the addition of MIL-101 (Cr) MOF into epoxy resin can significantly increase the heat release during the curing reaction of epoxy by 63% at a very low MOF loading of 0.1 wt.%. Such a significant rise in the cure enthalpy was rated as good and excellent according to the Cure Index [71,72], due to the large pore size of the MOF, having two internal cages with diameters of 29 Å and 34 Å which allow epoxy pre-polymer diffusion inside the 3D structure of the MOF, therefore accelerating the cross-linking by the aid of the Lewis acid sites of Cr that catalyzed epoxy ring opening (Figure 4). Similar behavior was observed for epoxy systems containing halloysite nanotubes (HNTs), which highlights the role of reactive groups in catalyzing epoxy ring opening [73,74,75].

Available reports are indicative of the fact that the incorporation of MOFs into an epoxy affects the ultimate properties of the epoxy, such as its anti-corrosion, flame-retardant, mechanical, and dielectric properties. Nevertheless, the mechanisms of the performance of MOFs in epoxy coatings should be further discussed due to the inadequacy of reports on this topic to date and also to make a possible demonstration of structure–property correlation in the MOF/epoxy coatings.

### 2.1. Anti-Corrosion Properties

In recent years, the anti-corrosion performance of MOFs in epoxy coatings has been at the center of attention. Diverse hydrophobic MOFs, such as ZIF-7, ZIF-8, MOF-5, and Ce-MOF, which are potentially favorable for anti-corrosion coatings, have been studied, as summarized in Table 1. 

The high-affinity interactions of MOFs with the inorganic and organic compounds allow them to form highly cross-linked epoxy/MOF composite coatings, which can further strengthen the anti-corrosion performance of the epoxy coatings. In addition, the ability to use different metal ions as well as organic linkers allows the creation of MOFs with pH, magnetic, molecular, thermo-, and pressure responsiveness [90]. Based on such characteristics, stimuli-responsive MOFs can intelligently respond to the environmental changes caused by the corrosion inhibitor nanocontainers in the epoxy coatings. Epoxy coatings filled with low amounts of such stimuli-responsive MOF nanocontainers not only provide the coatings with a barrier layer on the metal against the corrosive media (water, O_2_, and Cl^−^, etc.) but also protect the metal from further corrosion as a consequence of their corrosion-sensing and self-healing properties via controlling the amount of released corrosion inhibitors [91]. 

**Figure 5:**
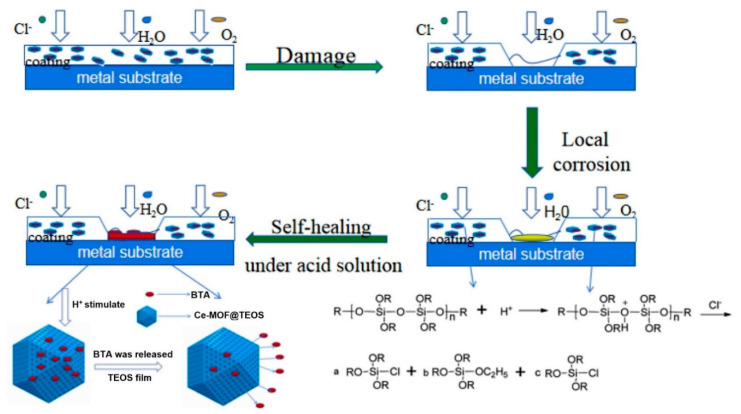
Schematic of the self-healing mechanism of the corrosion inhibitor encapsulated MOF/epoxy coatings [83].

Corrosive components attack the epoxy coatings on the metal substrates where defects and pores exist [92]. The presence of MOFs with excellent hydrophobicity in the epoxy matrix can improve the barrier properties of epoxy coatings by preventing the diffusion of corrosive ingredients into the coatings. However, epoxy/MOFs coatings are not able to provide long-lasting protection in the absence of corrosion inhibitors, and local corrosion may occur on the metal substrate, as schematically shown in Figure 5. In the case of epoxy coatings containing corrosion inhibitor encapsulated MOF, the corrosive medium rapidly permeates from the crack and attacks the corrosion inhibitor encapsulated MOF. Consequently, the corrosion inhibitor is released due to the pH-responsive nature of the MOFs’ coordination and the gradually adsorbed precursors on the metal surface. Finally, the inhibitor forms a film in the vicinity of the cracked zone to effectively prevent the cathodic reaction and further deterioration in a self-repairing manner.

### 2.2. Flame-Retardant Properties

The high flammability manifested by a high peak of heat release rate (pHRR), high value of total heat release (THR) in the cone calorimeter test as well as the dense smoke during the combustion of epoxy limits its applications [93,94]. Therefore, several research works have been dedicated to retarding the combustion and suppressing the amount of smoke released by epoxy by the use of halogen-free flame retardants. In recent years, MOF materials have been used in epoxy resins as a part of flame-retardant systems due to their super porous structures, and their chemistry as summarized in Table 2. 

Compared to the traditional inorganic nanoparticles, the benefits of MOFs spring from their compatibility and strong interactions with the epoxy matrix, without the need for further modification. Organic linkers in the structures of MOFs do not merely contribute to the compatibility of MOFs with the epoxy matrix but also contributes to flame retardancy due to nitrogen, or phosphorus-containing groups and aromatic rings [100]. In addition, the thermal degradation of MOFs results in the formation of transition metal oxides, which could have catalytic effect and facilitate char forming in the condensed phase [98]. Therefore, according to the structural features of MOFs, they can be used in flame retardant systems for enhancing the fire safety of epoxy coatings, an attractive alternative to the traditional nanoparticles.

**Figure 6:**
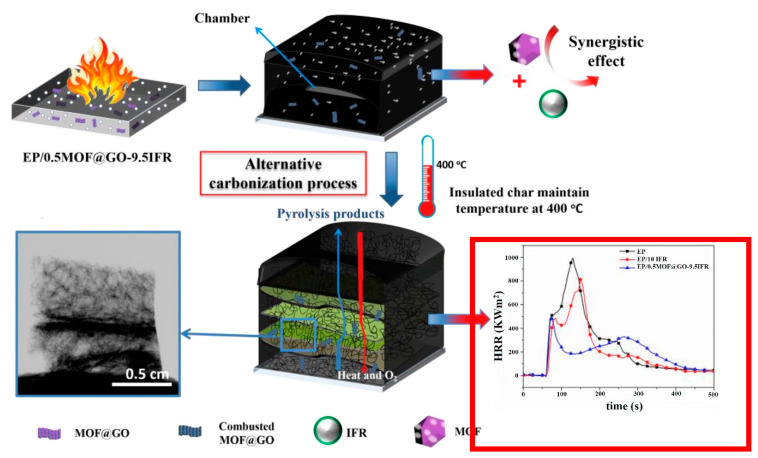
Fire retardancy mechanism of epoxy-containing phosphorus flame retardant loaded MOF [101].

Neat epoxy coatings release large amounts of heat, smoke, CO_2_ and CO in fire. It has been reported that the addition of MOFs can significantly decrease the release of heat, smoke, CO_2_, and CO in the epoxy matrix. Therefore, the flame-retardant mechanism of epoxy/MOF coatings can take place in both the gas and the condensed phases. MOFs can reduce the emission of released heat and CO formed during the decomposition of epoxy because of the catalytic oxidation effect of metal clusters in the MOF structure [99]. In the case of epoxy coatings containing phosphorus flame retardant loaded MOF, phosphorus compounds react with the active free radicals to restrict fire spread. Through a synergetic pathway, the effects of MOFs and phosphorus flame retardants in the condensed phase assist in the induction of the cross-linking in the epoxy condensed phase and the trapping of the degrading radicals which promote the carbonaceous process. This phenomenon leads to the formation of a compact barrier char, which prevents heat transfer and reduces the exposure of the epoxy matrix to the heat source, as presented in Figure 6 [101]. The effects of MOF nanoparticlessuggest their great potential for flame-retardant applications.

### 2.3. Other Properties

MOF/epoxy nanocomposites combine the high thermal stability of MOFs with the robustness of epoxy. Recent advances in this field are summarized in Table 3. Moreover, the brittleness and inadequate strength of the epoxy can be overcome by incorporating MOFs into the polymer matrix [102]. The superiority of the mechanical properties of MOF/epoxy nanocomposites is fueled by three phenomena taking place at a molecular level. Firstly, MOF nanoparticles participate in the curing reaction of epoxy through the Lewis acid sites and functional groups of organic linkers to increase the degree of cross-linking of the epoxy, which boosts its mechanical strength. Secondly, the polymer chains wrapped in the porous structures of MOFs act as dampers and help it to resist external stress. Thirdly, the large specific surface areas of porous MOFs enhance the interfacial region between the epoxy chains and the linkers at the atomic level much more than conventional nanoparticles [103]. The MOFs in the epoxy matrix act as elastic particles, which can be elongated or compressed depending on the MOF’s content and the force applied [104]. Moreover, MOFs can effectively absorb the mechanical energy and the dimensionality of the interaction between the metal ions and the organic ligands in their structure, which causes elastic transformations in specified directions, which in turn affect the MOF’s physical properties [105,106].

The new application of MOFs is in microelectronics, which draws researchers’ attention to the study of the electrical properties of MOFs. The ideal organic coatings for producing large scale integrated circuits should pass several requirements, such as low dielectric loss, high electrical insulation, good thermal stability, excellent mechanical properties, and low water absorption, in addition to having a low dielectric constant [110,111]. MOFs are useful as low-k materials because of their low polarity and density [112]. Moreover, MOFs possess a large specific surface area, porous structure, high thermal stability, resistance to chemicals, and excellent hardness [113,114]. Moreover, the introduction of porous MOFs into the epoxy matrix may decrease the density of the system by increasing the free volume in the cross-linked network. These features, as mentioned earlier, make MOFs promising low-κ dielectric materials for developing advanced epoxy coatings.

## 3. Future Ahead of MOFs for Coating Applications

MOFs can be considered as promising materials for epoxy coatings due to their versatile and tunable structures, large specific surface areas, low density, high capability to encapsulate active moieties, good ability for selective interactions, and the possibility for further functionalization. Although epoxy/MOF coatings have shown excellent anti-corrosion, flame retardancy, and mechanical performance, these nanocomposites should be comprehensively studied in order to tackle the challenges associated with their functionalization, dispersion in epoxy, or other kinds of thermoset resins, and their ability to encapsulate agents for corrosion inhibition purposes. The encapsulation of macromolecules such as polymers in MOFs is still a challenge that may cause agglomeration of the nanoparticles. By the design of organic ligands in the structures of MOFs, tailored MOFs can be synthesized for the broader application of coatings in the future. On the other hand, the quite facile synthesis route and the abundance of raw materials allow the MOFs to give rise to the possibility of producing industrial-scale nanocomposite coatings with a wide variety of properties. For instance, in the flame retardancy, more fundamental studies are needed to determine the role of integrated metals in MOFs in catalysis and the formation of char in the course of the combustion mechanism of epoxy coatings. The chemical modification/functionalization of MOFs by various oxidation degrees of phosphorus and their reaction with various integrated metals in MOFs during combustion may also provide useful information for future developments, but at the same time, they may bring about serious challenges. In conclusion, epoxy/MOF coatings constitute the next generation of multifunctional organic coatings that allow the detecting and sensing of defects in the matrix and the self-repairing of the damage. By the appropriate selection of MOF ligands and the modification of them with the functional groups with reactivity towards the epoxy matrix as well as using MOFs as capsules for corrosion inhibitors or flame-retardant materials, their future beyond these kinds of multifunctional epoxy coatings seems bright. It can be concluded that the availability of thousands of MOFs built from different cations, which are typically based on carboxylates, phosphonates or N donating linkers, or a combination of them in a wide range of structure types and pore sizes, from the micro to the meso domain (with or without functional groups), allows for the preparation of epoxy/MOF composites for advanced coating applications.

## Figures and Tables

**Table 1:** Epoxy/MOF nanocomposites applied as coatings, with satisfying anti-corrosion properties. The main outcomes of recent research are also provided. Different types of MOFs applied in coating technology are summarized in the following table, such as Cu-MOF [76] and ZIF-8/ZIF-7 [77,78]; Ce-MOFs [79], and MOF-5 [80] made of Cu (II) and Zn (II) as the nodes, and benzotriazole (BTA), 2-methylimidazole/benzotriazole (BIM), 2-methylimidazole, BTA, and terephthalic acid (H_2_BDC), respectively, as the organic bridges.

Epoxy/MOF Composite-Based Materials for Corrosion Inhibition					
No	MOFs	Corrosion Inhibitor	Corrosive Media	Main Findings	Reference
**1**	ZIF-8	Hollow mesoporous silica nanoparticles (HMSN)-BTA	3.5 wt.% NaCl solution	After 30 days of immersion in the corrosive solution, the values of film resistance (R_f_) of the epoxy coatings with HMSN-BTA@ZIF-8 were much higher than 108 Ω cm^2^, while values decreased to only about 106 Ω cm^2^ for the neat epoxy.	[81]
**2**	ZIF-8	Zinc Gluconate (ZnG)	3.5 wt.% NaCl solution	Incorporation of ZnG@ZIF-8 as a corrosion inhibitor revealed no signs of corrosion in the epoxy, even after 40 days of immersion.	[82]
**3**	Ce-MOF	BTA	3.5 wt.% NaCl solution	Incorporation of 3 wt.% BI loaded Ce-MOF@ tetraethyl orthosilicate (TEOS) into the epoxy coating improved the charge transfer resistance by 1.5% after 2 h of immersion.	[83]
**4**	ZIF-7	BTA	0.1 M HCl solution	The epoxy coating doped with ZIF-7@BTA exhibited a superior barrier performance, which provided 99.4% inhibition efficiency.	[84]
**5**	Cu-MOF	BTA	3.5 wt.% NaCl solution	Incorporation of 2 wt.% BTA-Cu-MOF into epoxy exhibited a corrosion potential of 0.492 V, which was ca. 26.5 times the value of the polarization resistance of the blank epoxy.	[85]
**6**	ZIF-7	-	0.1 M HCl solution	The coating resistance of 1.7% ZIF incorporated epoxy was ca. 2 times of that of the blank epoxy after 72 h of immersion.	[86]
**7**	MOF-5	-	3.5 wt.% NaCl solution	Dopamine@MOF-5 effectively delayed penetration of corrosive solution into the coating for 480 h.	[87]
**8**	ZIF-8	-	5 wt.% NaCl solution	RHS *@ZIF-8 structure improved early-stage corrosion inhibition of epoxy with no essential damage in the coating.	[88]
**9**	ZIF-8	-	3.5 wt.% NaCl solution	By addition of graphene oxide (GO)@ZIF-8, the cathodic delamination resistance, and wet adhesion strength were improved by about 73% and 60%, respectively.	[89]

* 3,6-Bis(diethylamino)-2-[[(2-hydroxyphenyl)methylene]amino]spiro [1H-isoindole-1,90-[9H]xanthen]-3(2H)-one.

**Table 2:** Epoxy/MOF nanocomposites applied in the FR systems. The main outcomes of recent research are also provided. Different types of MOFs are collected in the following table, such as ZIF-67; NH2-MIL101 (Al), Co-MOF [95] and MIL-101 (Fe) [96] are made of Zn (II), Al (III) and Fe (III) as the nodes and, BTA, NH_2_- H2BDC, 1,4-benzodicarboxylic acid, di (para-aminobenzoic acid) phenyl phosphate amide, and terephthalic acid, respectively, as the organic bridges.

Epoxy-MOF Composite-Based Materials for Flame Retardancy					
No	MOFs	Flame Retardant	Flame Tests	Main Findings	Reference
**1**	Co-MOF	Di (para-aminobenzoic acid) phenyl phosphate amide	CC ^1^ SSTF ^2^	Decrease in pHRR and THR by 28% and 18.6%, respectively, by incorporating 2 wt.% of phosphorus-Co-MOF.	[97]
**2**	ZIF-8 ZIF-67 MIL-101 (Fe)	-	CC	2 wt.% of Co-MOF/epoxy, Zn-MOF/epoxy, and Fe-MOF/epoxy composites burnt relatively slowly, and the reduction in pHRR in the composites was 31.3%, 27.8%, and 18.6%, respectively.	[98]
**3**	NH_2_-MIL-101 (Al)	Phosphorus-nitrogen-containing ionic liquid (IL@NH_2_)	LOI ^3^ CC	Addition of 3 wt.% IL modified-MOF (IL@NH_2_-MIL-101 (Al)) increased the LOI value of the epoxy to 29.8%, decreased pHRR by 51.2%, smoke production rate by 37.8%, and CO release rate by 44.8% with respect to those of blank epoxy.	[99]

^1^ Cone calorimeter (CC); ^2^ Steady state tube furnace (SSTF); ^3^ Limiting oxygen index (LOI).

**Table 3:** Epoxy/MOF nanocomposites applied as coatings with satisfying mechanical and dielectric properties. The main outcomes of recent research are also provided.

MOFs as the Modifiers of Other Properties			
No	MOFs	Main Findings	Reference
**1**	MOF-5	Incorporation of 0.3% wt. MOF-5 led to 68% rise in the impact strength and 230% increase in the fracture energy of epoxy.	[104]
**2**	UiO-66 * UiO-66-NH_2_	The values of tensile strength and elongation at break for UiO-66-NH_2_/EP were 40.4 MPa and 2.60%, respectively, which were higher than those of neat epoxy (35.2 MPa and 1.94%, respectively) and UiO-66/epoxy (37.0 MPa and 2.56%, respectively).	[103]
**3**	ZIF-8	Addition of 25 vol.% ZIF-8 increased the Young’s modulus by 20% and decreased the dielectric constant of epoxy from 3.9 to 3.2 at 100 kHz.	[107]
**4**	ZIF-8	The dielectric constant of the epoxy/ZIF-8 composite (0.3 wt.%) was decreased from 4.12 to 3.45.	[108]

* Uio-66 [109] is made of Zr (II) and 1,4-benzodicarboxylic acid as the organic bridges.
